# Maternal cigarette smoking and its effect on neonatal lymphocyte subpopulations and replication

**DOI:** 10.1186/1471-2431-13-57

**Published:** 2013-04-18

**Authors:** Giovanni Almanzar, Gernot Eberle, Andrea Lassacher, Christian Specht, Christian Koppelstaetter, Peter Heinz-Erian, Rudolf Trawöger, David Bernhard, Martina Prelog

**Affiliations:** 1 Department of Pediatrics, University of Wuerzburg, Josef-Schneider-Str. 2, 97080 Wuerzburg, Germany; 2 Department of Pediatrics, Medical University of Innsbruck, Anichstr. 35, Innsbruck, Austria; 3 Department of Gynecology and Obstetrics, Medical University of Innsbruck, Anichstr. 35, Innsbruck, Austria; 4 Department of Internal Medicine, Medical University of Innsbruck, Anichstr. 35, Innsbruck, Austria; 5 Cardiac Surgery Research Laboratory, Cardiac Surgery, Medical University of Innsbruck, Innsbruck, Austria

**Keywords:** Cigarette smoking, Lymphocytes, T cell receptor excision circles, Telomeres

## Abstract

**Background:**

Significant immunomodulatory effects have been described as result of cigarette smoking in adults and pregnant women. However, the effect of cigarette smoking during pregnancy on the lymphocyte subpopulations in newborns has been discussed, controversially.

**Methods:**

In a prospective birth cohort, we analyzed the peripheral lymphocyte subpopulations of smoking (SM) and non-smoking mothers (NSM) and their newborns and the replicative history of neonatal, mostly naive CD4 + CD45RA + T cells by measurements of T-cell-receptor-excision-circles (TRECs), relative telomere lengths (RTL) and the serum cytokine concentrations.

**Results:**

SM had higher lymphocyte counts than NSM. Comparing SM and NSM and SM newborns with NSM newborns, no significant differences in proportions of lymphocyte subpopulations were seen. Regardless of their smoking habits, mothers had significantly lower naive T cells and higher memory and effector T cells than newborns. NSM had significantly lower percentages of CD4 + CD25++ T cells compared to their newborns, which was not significant in SM. There were no differences regarding cytokine concentrations in newborns of SM and NSM. However, NSM had significantly higher Interleukin-7 concentrations than their newborns. Regardless of smoking habits of mothers, newborns had significantly longer telomeres and higher TRECs than their mothers. Newborns of SM had significantly longer telomeres than newborns of NSM.

**Conclusions:**

Apart from higher lymphocyte counts in SM, our results did not reveal differences between lymphocyte subpopulations of SM and NSM and their newborns, respectively. Our finding of significantly longer RTL in newborns of SM may reflect potential harm on lymphocytes, such as cytogenetic damage induced by smoking.

## Background

Significant alterations of the lymphocyte proportions [1,2] and higher inflammatory cytokines, such as IL-1β, IL-4, IL-6 and TGF-β [3-5], and lower TNF-α, IL-2 and IFN-γ concentrations [6] have been described as result of cigarette smoking in healthy individuals [7]. Smoking during pregnancy has been shown to be an independent risk factor for respiratory diseases in children [8]. For pregnant women, increase of NK cells and inflammatory macrophages, as well as lower percentages of regulatory T cells (Tregs) have been reported, with lower birth weights in their neonates [9]. Most knowledge about the role of cigarette smoking on the composition of the different lymphocyte subpopulations in neonates has been generated from studies in mice, in which a controlled exposure to a defined component of tobacco smoke was performed. A significant reduction of newborn CD4 + CD8+, CD4 + CD8+ V beta 8+ thymocytes and CD4+ splenocytes from 1-week-old progeny was shown in mice exposed to benzo[a]pyrene, a critical component of cigarette smoke [10]. In a study investigating 14 newborns of smoking mothers, significantly lower leukocyte counts with a most prominent decrease in segmented neutrophils, monocytes and lymphocytes were found compared to 74 newborns of non-smoking mothers [11].

According to results from adult smokers, pregnant smoking women and prenatally cigarette-smoke exposed newborns, our hypothesis was that components of cigarette smoke may cause distress on fetal lymphocytes, resulting in higher differentiation of T cell subpopulations, less naive T cells, a higher replicative history of peripheral T cells, as measured by T cell receptor excision circles (TRECs) [12,13] and relative telomere length [14], and higher pro-inflammatory cytokines, such as IL-1β, IL-6 or TNF-α. IL-7 is seen as a cytokine inducing peripheral naive T cell proliferation.

Thus, the present, prospective birth cohort was aimed to analyze the peripheral lymphocyte subpopulations of smoking (SM) and non-smoking mothers (NSM) and their newborns, the replicative history of neonatal, mostly naive CD4 + CD45RA + T cells and the serum cytokine concentrations.

## Methods

### Study participants

Smoking (SM) and non-smoking mothers (NSM) and their newborns were recruited at the Department of Gynecology and Obstetrics, Medical University Innsbruck, Austria, after giving written informed consent. The study was approved by the local ethics committee and was performed according to the principles of good clinical and laboratory practice and the Declaration of Helsinki.

Only Caucasian mothers were included who were healthy without underlying medical conditions or medications and who had uncomplicated, spontaneous vaginal delivery of a healthy, mature newborn (showing at least 5 maturity signs according to [15]) between gestational weeks 37 and 42. The SM group was defined by both a history of cigarette smoking during pregnancy and by positive cotinine concentrations >25 ng/ml from venous blood sampling at admission to the maternity ward (2 to 24 hours before birth). The mothers were asked about their smoking behavior using a standardized questionnaire, including the number of smoked cigarettes before and during pregnancy and the brand and nicotine strength of the smoked filter-tipped or non-filter cigarettes. The addictive behavior was controlled by Fagerström test for nicotine dependence questionnaire [16]. Maternal exclusion criteria were: atopy, autoimmune disorders, malignancies, chronic organ insufficiency, drugs, alcohol during pregnancy, medications, high blood pressure (systolic blood pressure >150 mmHg), eclampsy, diabetes during pregnancy, positive bacterial vaginal swab in the last 3 weeks before delivery, antibiotics in the last 3 weeks before delivery or during delivery, vacuum extraction and passive smoking in the NSM group.

Neonatal exclusion criteria were: pathological cardiotocogram, APGAR score < 9 in the first minute, bradycardia (< 100/minute), tachydyspnea (frequency breath taking >60/min), signs of infection or fetal distress (e.g. green-stained amniotic fluid) and congenital syndromes. Cord blood was collected between 1 and 5 minutes after birth.

### Lymphocyte isolation and characterization

Lymphocytes from venous blood or cord blood were isolated using Biocoll Separating Solution (Biochrom AG, Berlin, Germany). Lymphocyte subpopulations were measured by fluorochrom-labeled antibodies (all purchased from BD Pharmigen, San Jose, CA, USA) with flowcytometry according to following definitions: CD3+ (T cells), CD19+ (B cells), CD16+/CD56+ (NK cells), CD45RA + CD28 + CD62L + (naive T cells), CD45RO + CD27 + CD28+ (early memory T cells), CD45RO + CD27-CD28- (late memory T cells), CD45RA + CD28- (effector T cells), CD4 + CD25++ (bright) (representing features of naive T cells, regulatory T cells, Tregs, and activated T cells). Results were expressed as percentages of gated lymphocytes.

### Quantification of TRECs and relative telomere length

DNA was extracted from separated CD4^+^CD45RA^+^ T cells using QIAamp DNA Mini Kit (Qiagen, Chatsworth, CA, USA). Signal-joint TREC concentrations were determined by quantitative SYBR-green real-time PCR based on the coding TREC sequence using an iCycler quantitative RT-PCR system (BioRad Laboratories, Hercules, Canada) and log2 dilutions of an internal standard as described previously [12,17]. To avoid bias by different numbers of naive T cells, TRECs were calculated in relation to CD4 + CD45RA + T cell numbers [13].

Determination of relative telomere length (RTL) was performed by calculating the ratio of a quantitative PCR reaction product from the same sample using specific primers for telomeres and a single copy gene as described previously [14,18].

### Cytokine measurements

ELISAs for measurements of IL-1β, IL-2, IL-4, IL-6, IL-7, IL-8, IL-10 and TNF-α were performed according to the manufacturer’s instructions (R&D Systems Europe, Minneapolis, MN, USA).

### Cotinine concentrations

Cotinine concentrations in serum from maternal venous blood and cord blood were measured using a commercial ELISA (DRG International, Marburg, Germany). Cotinine concentrations higher than 25 ng/ml were defined to be positive.

### Statistics

Distribution of data was tested by Kolmogorov-Smirnov test using SPSS Version 19.0 (Chicago, IL, USA). Normally distributed data were analyzed with student’s *T* test, not normally distributed data with Mann–Whitney-*U* test. Multiple linear regression analysis was performed in search for independent variables, including maternal smoking, age of mother at birth, gestational week, male or female gender, birth weight, birth length, head circumference and APGAR at 1 and 5 minutes. A p-value < 0.05 was considered to be statistically significant.

## Results

### Epidemiological characteristics of mothers and their newborns

There was no significant difference between non-smoking mothers (NSM) and smoking mothers (SM) regarding age (Table 1). SM reduced their cigarette consumption during pregnancy to 61.5% in the first, 57.0% in the second and 58.5% in the third trimenon. The mean birthweight of SM newborns was significantly lower than that of their NSM counterparts (Table 1). SM had more male newborns (63.8%) than NSM (54.1%) (Table 1).

**Table 1:** Epidemiological characteristics of mothers and newborns

	**Non-smoking mothers (NSM) (n = 111)**	**Smoking mothers (SM) (n = 58)**
**Maternal age at birth (years)**	31.1 ± 5.7 (31.2; 16.0 – 46.4)	29.0 ± 6.0 (28.2; 18.5 – 40.4)
**Cigarettes/day before pregnancy**	(n = 10) 11.3 ± 7.3 (12.0; 1.5 -20.0)	(n = 58) 20.0 ± 9.3 (20.0; 2.0 – 40.0)***
**Years of tobacco use**	9.0 ± 4.4 (10.0; 2.0 – 20.0)	11.5 ± 5.7 (10.0; 2.0 – 24.0)
**First trimenon (cigarettes/day)**	0	12.3 ± 7.1 (10.0; 2.0 – 30.0)
**Second trimenon (cigarettes/day)**	0	11.4 ± 6.8 (10.0; 2.0 – 30.0)
**Third trimenon (cigarettes/day)**	0	11.7 ± 7.3 (10.0; 1.0 – 30.0)
**Sex of newborn (male/female) (number)**	60/51	37/21
**Gestational age (weeks)**	39.6 ± 1.4 (39.1; 37.3 – 41.7)	39.5 ± 1.5 (39.4; 37.1 – 41.7)
**Birth weight (g)**	3379.3 ± 429.9 (3380.0; 2330.0 – 4355.0)	3118.2 ± 497.7 (3040.0; 2190.0 – 4190.0)***^1)^
**Birth length (cm)**	50.4 ± 2.0 (51.0; 46.0 – 57.0)	49.1 ± 2.7 (49.0; 43.0 – 55.0)*** ^1)^
**Head circumference (cm)**	35.0 ± 1.3 (35.0; 32.0 – 38.0)	34.3 ± 1.2 (35.0; 32.0 – 37.0)* ^2)^

Values are given in mean ± standard deviation (median; range). Numbers of subjects, n.

Statistical differences (non-parametric Mann–Whitney-*U* test):

*** p < 0.001: higher than NSM value.

Statistical difference (parametric Student’s *T* test):

***^1)^ p < 0.001: lower than NSM values.

* ^2)^ p < 0.05: lower than NSM value.

Of SM, 24 smoked light cigarettes (nicotine content between 0.6 to 1.0 mg), 31 smoked normal cigarettes (nicotine content between 1.2 to 1.4 mg) and 3 smoked light and normal cigarettes. The mean maternal cotinine concentrations were 42.6 ± 7.0 ng/ml. The mean neonatal cotinine concentrations were 42.9 ± 6.6 ng/ml. Fagerström questionnaire revealed 2 to 5 points (moderate addiction) in 35 SM, 6 to 11 points (strong addiction) in 23 SM.

### Blood cell counts and percentages of lymphocyte subpopulations

SM had significantly lower platelet counts (182.8 ± 46.3 G/l) than NSM (229.9 ± 47.1 G/l), but higher lymphocyte counts (Table 2). There were no significant differences in red blood cell counts between newborns of SM and NSM.

**Table 2:** Lymphocyte subpopulations in newborns and non-smoking (NSM) and smoking mothers (SM)

	**Newborns**		**Mothers**	
	**NSM (n = 111)**	**SM (n = 58)**	**NSM (n = 111)**	**SM (n = 58)**
**Leucocytes/μl**	11948 ± 3771 (11340; 5440 – 23980)	11285 ± 2796 (11600; 4570 – 16810)	11863 ± 5040 (11180; 4140 – 25510)	13404 ± 4390 (12700; 6980 – 21520)
**Lymphocytes/μl**	4084 ± 1263 (3800; 1580 – 8500) ***	4046 ± 1057 (3930; 2320 – 6100)***	1277 ± 493 (1250; 480 – 2660)	1897 ± 722 (1810; 900 – 4260) ***^1)^
**CD3+ (% of lymphocytes)**	69.2 ± 8.8 (69.4; 51.6 – 86.7)	68.8 ± 8.7 (68.3; 50.5 – 90.3)	80.1 ± 6.9 (80.8; 56.6 – 91.6)	79.8 ± 6.9 (80.3; 62.1 – 91.6)
**CD19+ (% of lymphocytes)**	16.9 ± 6.2 (15.2; 5.0 – 35.1)	18.2 ± 4.9 (18.4; 8.6 – 26.3)	10.2 ± 4.3 (9.3; 2.8 – 24.4)	12.1 ± 5.5 (11.5; 4.6 – 27.5)
**CD16+/56+ (% of lymphocytes)**	13.8 ± 8.5 (11.4; 3.0 – 39.5)	13.1 ± 7.6 (12.5; 1.1 – 26.5)	9.7 ± 7.0 (7.8; 1.9 – 38.6)	8.1 ± 4.7 (7.6; 1.7 – 25.1)
**CD4/CD8**	2.5 ± 0.9 (2.4; 0.9 – 5.0)	2.4 ± 0.7 (2.4; 1.3 – 3.8)	1.7 ± 0.6 (1.6; 0.7 – 3.2)	1.8 ± 0.6 (1.7; 0.9 – 3.9)
**CD4+ (% of CD3+)**	69.7 ± 8.1 (70.8; 48.8 – 83.4)	69.5 ± 6.0 (70.1; 56.3 – 79.3)	61.2 ± 8.0 (61.5; 41.9 – 75.9)	61.9 ± 7.9 (63.3; 46.4 – 79.8)
**CD8+ (% of CD3+)**	30.4 ± 8.1 (29.2; 16.6 – 51.2)	30.5 ± 6.1 (29.8; 20.7 – 43.8)	38.8 ± 8.0 (38.5; 23.9 – 58.0)	38.0 ± 7.9 (36.7; 20.3 – 53.6)
**CD45RA + CD28 + CD62L + (% of CD4+)**	85.3 ± 8.9 (85.4; 48.4 – 94.8)	86.7 ± 6.6 (87.5; 70.8 – 98.6)	45.1 ± 14.1 (45.6; 19.5 – 62.4) ***^2)^	42.4 ± 12.1 (44.1; 20.6 – 67.2) ***^2)^
**CD45RA + CD28 + CD62L + (% of CD8+)**	59.3 ± 12.9 (58.9; 20.5 – 85.7)	60.5 ± 10.9 (59.1; 31.6 – 77.3)	38.8 ± 17.6 (36.9; 12.0 – 83.3) ***^2)^	37.4 ± 11.5 (38.8; 11.2 – 59.0) ***^2)^
**CD45RO + CD27 + CD28+ (% of CD4+)**	4.9 ± 2.4 (4.7; 0.5 – 11.9) ***^3)^	4.7 ± 1.9 (4.8; 0.6 – 9.2) ***^3)^	34.3 ± 10.6 (33.1; 14.2 – 65.2)	37.1 ± 8.5 (38.5; 17.6 – 51.0)
**CD45RO + CD27 + CD28+ (% of CD8+)**	3.4 ± 1.7 (3.4; 0.0 – 9.3) ***^3)^	3.1 ± 1.8 (3.1; 0.0 – 7.2) ***^3)^	13.4 ± 7.6 (11.8; 3.2 – 49.1)	12.1 ± 5.8 (10.4; 3.7 – 24.5)
**CD45RO + CD27-CD28-(% of CD4+)**	0.3 ± 0.5 (0.1; 0.0 – 2.5) ***^3)^	0.4 ± 0.5 (0.1; 0.0 – 2.0) ***^3)^	2.1 ± 3.1 (0.7; 0.1 – 18.1)	1.5 ± 2.4 (0.6; 0.1 – 11.1)
**CD45RO + CD27-CD28-(% of CD8+)**	0.3 ± 0.7 (0.1; 0.0 – 4.3) ***^3)^	0.2 ± 0.4 (0.0; 0.0 – 2.0) ***^3)^	4.0 ± 6.4 (2.5; 0.1 – 22.0)	2.8 ± 3.5 (1.8; 0.1 – 18.6)
**CD45RA + CD28- (% of CD4+)**	0.05± 0.2 (0.0; 0.0 – 1.7) ***^3)^	0.02 ±0.04 (0.0; 0.0 – 0.1) ***^3)^	1.0 ± 1.8 (0.2; 0.1 – 7.8)	0.9 ± 2.4 (0.2; 0.1 – 11.9)
**CD45RA + CD28- (% of CD8+)**	3.3 ± 4.9 (2.4; 0.3 – 4.8) ***^3)^	2.2 ± 1.1 (2.3; 0.1 – 6.1) ***^3)^	16.9 ± 12.9 (14.6; 0.4 – 57.9)	16.7 ± 11.9 (13.8; 1.0 – 40.9)
**CD4 + CD25++ (% of CD3+)**	6.1 ± 2.9 (6.6; 0.0 – 11.7)	6.0 ± 3.3 (7.3; 0.0 – 10.7)	5.4 ± 3.9 (5.2; 0.1 – 17.0)*^4)^	6.2 ± 4.7 (6.1; 0.1 – 17.7)

Values are given in mean ± standard deviation (median; range). Numbers, n.

Statistical differences (parametric Student’s *T* test):

*** p < 0.001: higher than respective maternal values.

Statistical differences (non-parametric Mann–Whitney-*U* test):

***^1)^ p < 0.001: higher than NSM value.

***^2)^ p < 0.001: higher than respective newborn values.

***^3)^ p < 0.001: lower than respective maternal values.

*^4)^ p < 0.05: lower than respective newborn value.

Comparing the whole groups of SM and NSM, no significant differences in proportions of lymphocyte subpopulations in mothers or newborns were seen (Table 2). There were no differences between the subgroups of SM smoking light or normal cigarettes and between SM with moderate and strong addiction according to Fagerström questionnaire. Regardless of their smoking habits, mothers had significantly lower proportions of naive T cells, higher proportions of memory T cells, of early and late memory T cells and of effector T cells than their newborns. NSM had significantly lower percentages of CD4 + CD25++ T cells compared to their newborns, which was not significant in SM.

Analysis of the data of the subjects of the NSM group who smoked prior to pregnancy and comparison of these subjects to the SM group who continued smoking during pregnancy and the NSM group who never smoked did not reveal any differences.

### Cytokine concentrations

**Figure 1:**
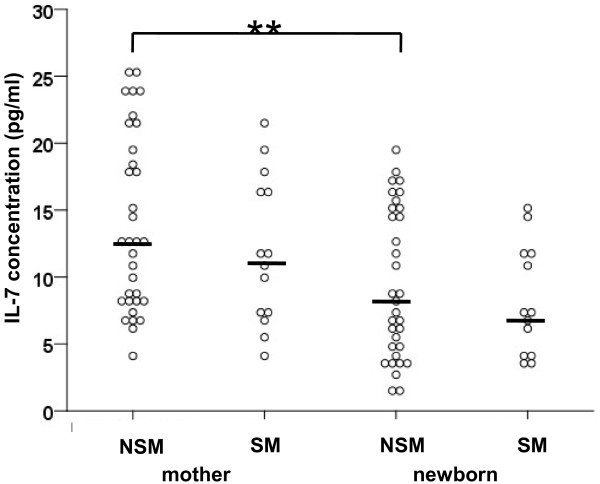
**Interleukin-7 (IL-7) concentrations in non-smoking (NSM) and smoking mothers (SM) and newborns.** NSM had significantly higher IL-7 concentrations than their newborns (** p < 0.01). Non-parametric Mann–Whitney-*U* test.

There were no significant differences regarding cytokine concentrations in newborns of SM and NSM (Table 3). However, NSM had significantly higher IL-7 concentrations than their newborns (Figure 1). Also, SM showed a trend to higher IL-7 concentrations compared to their newborns (p = 0.07) (Figure 1).

**Table 3:** Cytokine concentrations in newborns of non-smoking (NSM) and smoking mothers (SM)

	**Newborns**	
**pg/ml**	**NSM (n = 33)**	**SM (n = 13)**
**IL-1β**	87.5 ± 98.2 (52.0; 14.1 – 265.4)	87.6 ± 13.8 (80.8; 78.6 – 103.5)
**IL-2**	44.6 ± 51.1 (25.1; 5.0 – 91.9)	53.3 ± 14.0 (53.5; 22.1 – 73.7)
**IL-4**	26.2 ± 22.7 (16.2; 1.6 – 76.3)	18.8 ± 6.7 (17.5; 12.3 – 28.0)
**IL-6**	112.5 ± 204.8 (28.3; 0.6 – 737.8)	241.3 ± 408.2 (36.0; 7.4 – 1037.5)
**IL-8**	685.3 ± 1141.8 (166.4; 6.1 – 4474.5)	879.4 ± 1361.3 (41.6; 0.2 – 4352.4)
**IL-10**	6.3 ± 9.3 (2.9; 0.6 – 40.7)	3.5 ± 2.8 (2.7; 0.6 – 8.8)
**IFN-γ**	4.9 ± 3.6 (4.8; 0.1 – 12.8)	5.2 ± 3.2 (5.1; 1.1 – 9.8)
**TNF-α**	8.1 ± 8.7 (3.9; 1.3 – 29.2)	19.3 ± 32.6 (3.9; 1.3 – 89.1)

Values are given in mean ± standard deviation (median; range). Numbers, n.

### TRECs and RTL

**Figure 2:**
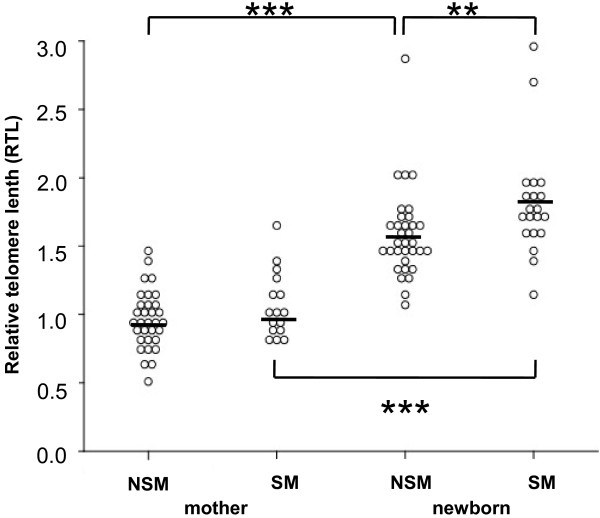
**Relative telomere length (RTL) in non-smoking (NSM) and smoking mothers (SM) and newborns.** Newborns of SM had significantly longer telomeres than newborns of NSM (** p < 0.01). Regardless of smoking habits, mothers had shorter telomeres than their respective newborns (*** p < 0.001). Non-parametric Mann–Whitney-*U* test.

Regardless of smoking habits of mothers, newborns had significantly higher TREC numbers and longer telomeres than their mothers (Table 4). Comparing RTLs of newborns, newborns of SM had significantly longer telomeres than newborns of NSM (Figure 2). In multiple regression analysis, adjustment for age of mother at birth, gestational week, gender, birth weight, birth length, head circumference and APGAR at 1 and 5 minutes did not alter these results.

**Table 4:** T cell receptor excision circles (TRECs) in non-smoking (NSM) and smoking mothers (SM) and their newborns

	**Newborns**		**Mothers**	
	**NSM (n = 16)**	**SM (n = 11)**	**NSM (n = 16)**	**SM (n = 11)**
**TRECs/1000 CD4 + CD45RA + cells**	103 ± 125 (69; 16 – 589)***	166 ± 269 (55; 11 – 985)***	32 ± 31 (22; 4 – 146)	51 ± 58 (20; 7 – 193)

Values are given in mean ± standard deviation (median; range).

Statistical difference (non-parametric Mann–Whitney-*U* test):

***p < 0.001 higher than respective maternal values.

## Discussion

Our prospective birth cohort study demonstrated no significant influence of maternal cigarette smoking on the percentages of lymphocyte subpopulations in newborns. This was surprising as we expected alterations of the proportions of lymphocytes in newborns of SM with higher replication rates of peripheral naive T cells, estimated by TRECs and RTL, which was thought to be caused by a pro-inflammatory potential of cigarette smoke components. However, with the exception of longer RTLs in newborns of SM, neither lymphocyte subpopulations, nor TRECs or cytokine concentrations were significantly different between newborns of SM and NSM.

Our findings were in contrast to a study showing significantly lower leukocyte counts with a most prominently decrease in segmented neutrophilic granulocytes, monocytes and lymphocytes in neonates of smoking mothers [11]. In that study, number of smoking mothers was small (14 versus 74 non-smoking mothers) and ways of delivery differed. In that study, neonatal distress (e.g. meconium within amniotic fluid, postnatal supplemention of oxygen), bacterial colonization or inflammation (e.g. positive vaginal smear, premature rupture of membranes) were not excluded, which may influence the investigated parameters. However, our study confirmed the previously found decrease in birth weight associated with maternal smoking [19].

According to literature [12,20], mothers showed significantly lower TRECs and shorter telomeres than their neonates. Interestingly, newborns of SM demonstrated longer telomeres than newborns of NSM. Telomere lengths reflect the replicative history of each individual cell, which implicates a lower replicative rate in CD4 + CD45RA + T cells, in part also supported by a trend to higher TREC numbers in newborns of SM. A recent study showed significant cytogenetic damage of lymphocytes with changes of the mitotic index in neonates of SM [21]. This is in agreement with the finding in our study of less replication of CD4 + CD45RA + T cells, as suggested by longer RTLs in this lymphocyte subpopulation. In neonates, CD4 + CD45RA + T cells may represent the naive T cell pool. In adults, not only naive T cells, but also CD45RA + CD28- effector T cells contribute to the CD4 + CD45RA + T cell pool [22]. Usually in adults, these CD45RA + effector T cells are clonally exhausted and are negative for TRECS with short telomeres. However, CD45RA-expressing effector T cells can be neglected in newborns. Short telomere lengths have been associated with decreased lung function and increased risk of chronic obstructive pulmonary disease in adult smokers, with the association markedly attenuated after adjusting for age and other confounders [23]. No effect of smoking on RTL was seen in SM compared to NSM in our groups. However, the mothers were younger than in that study [23] and had no history of an impaired lung function or chronic obstructive pulmonary disease. Additionally, as demonstrated by that study [23], several parameters confound telomere lengths in adults, thus, in contrast to the findings in newborns, making also our results from mothers difficult to interpret.

Cytokines were not altered in newborns of SM compared to NSM. Cytokine responses to allergens were tested in a study investigating newborns whose mothers smoked throughout pregnancy [24]. In that study, higher but only partly significant responses for IL-13, IL-9, IL-5 and IL-6 were seen in newborns of smoking mothers, with no difference in IFN-γ responses. Similar to that study, the large variation in serum cytokines may not allow to draw clear effects of maternal smoking on newborn cytokine production in our cohort. SM and NSM showed higher IL-7 concentrations compared to their newborns, which may reflect the suggested role of IL-7 for proliferation of peripheral naive T cells to maintain the peripheral T cell pool in adults [25,26].

Interestingly, SM had higher absolute lymphocyte numbers, which was also shown by others [27]. In contrast to other studies [27,28], proportions of CD3+ [9], CD4+ and CD8+ T cells, with an emphasis of CD4+ memory T cells [29], and B cells were not increased, as well as CD4 + CD25++ T cells were not decreased in SM in our study. Influences of dose, duration of smoke exposure and the ethnic background have been suggested to cause the heterogeneity in the findings [30], as well as redistribution of specific lymphocyte subpopulations from the periphery to other compartments, e.g. the bronchial lining associated with cigarette smoking [31,32]. CD4 + CD25++ T cells do not necessarily consist of Tregs, but contribute also to the naive and activated T cell pool. Thus, for comparability with other studies [9], Foxp3 should be included in further studies to define truly regulatory T cells. Overall, no difference in the investigated lymphocyte subpopulations was seen between SM and NSM. These findings are similar to a study investigating acute effects of smoking and environmental tobacco smoke in five smokers and non-smokers [33], showing increased NK cells and decreased CD3+ and CD19+ cells which did not reach statistical significance. However, our results may be limited by the markers used to define the different lymphocyte subpopulations. Possibly, more differences between newborns of SM and NSM would have been revealed by investigating markers which are linked to more functional features of lymphocytes, such as expression of chemokine receptors, activation markers and intracellular cytokine production.

One strength of our study is the strict inclusion criteria, including only vaginally delivered term newborns with full maturity signs and no pre- or perinatal distress, to exclude changes of lymphocyte subpopulations caused by different gestational age, maturity or potential inflammatory mechanisms [34]. However, one drawback of our investigation remains, that serum cotinine concentrations rather reflect acute exposure to cigarette smoke than its long term effects. Thus it could be that women, who did not smoke for a few days immediately before birth may have had inappropriately low serum cotinine concentrations whereas women passively or actively exposed during that time exhibited peak cotinine levels. Because of this and the known uncertainties of patient history taking, future studies should try to more adequately determine “true” cigarette smoke exposure, for example by measuring cotinine concentrations in the hairs from mothers and newborns [35].

## Conclusions

Thus, although our results did not reveal striking differences between newborns of SM and NSM, our finding of significantly increased RTLs in smoke-exposed newborns may be an important indication that cigarette smoking may cause severe cytogenetic damage in lymphocytes [21]. Also, expected long-term effects of maternal smoking during pregnancy on the cytokine profile and inflammatory potential in their offspring should cause increased awareness.

## Competing interests

The authors declare that they have no competing interests.

## Authors’ contributions

GA performed the interpretation of the flowcytometry data, contributed to the preparation of the manuscript and critical discussion of the results. GE recruited the patients, performed the isolation of the lymphocyte subpopulations and the flowcytometry. AL recruited the patients, prepared the DNA and performed the TREC analysis. CS recruited the patients, collected and interpreted the clinical data of NSM and SM. CK performed the telomere length analysis and interpreted the RTL data. PH-E performed the cotinine measurements, contributed to the preparation of the manuscript and critical discussion of the results. RT recruited the patients, collected and interpreted the clinical data of newborns. DB performed the analysis of cytokines. MP designed the study and wrote the manuscript. All authors read and approved the final manuscript.

## Pre-publication history

The pre-publication history for this paper can be accessed here:

http://www.biomedcentral.com/1471-2431/13/57/prepub
